# Seed priming and abiotic stress tolerance in carrot: Unraveling the mechanisms of improved germination

**DOI:** 10.1371/journal.pone.0318753

**Published:** 2025-02-07

**Authors:** Marcin Nowicki, Marzena Nowakowska, Katarzyna Nowak, Wojciech Szczechura, Piotr Kaminski

**Affiliations:** 1 Department of Entomology and Plant Pathology, University of Tennessee, Knoxville, Tennessee, United States of America; 2 Department of Horticultural Crop Breeding, The National Institute of Horticulture, Skierniewice, Poland; Arish university, Faculty of agricultural and environmental sciences, EGYPT

## Abstract

Climate change necessitates the development of improved crops capable of withstanding future weather patterns. Carrots (*Daucus carota* L.), a crucial vegetable crop of global importance, face unique challenges in seed germination and seedling development due to their complex pollination biology and outcrossing reproduction mode with severe inbreeding depression if selfed. This study investigated the effects of salinity and drought stress on carrot seed germination and seedling development, with focus on the roles of seed priming, cellular processes inhibitors, and biochemical responses. Seed priming agents were hypothesized to enhance stress tolerance by modulating specific cellular and biochemical pathways, such as improving osmotic balance, enhancing antioxidant defense mechanisms, and activating stress-responsive genes. We also hypothesized that specific cellular processes and biochemical pathways influence the germination and early seedling growth of carrot seeds under salinity or drought stress. To test that hypothesis, we evaluated the effects of seed priming with various agents (e.g., water, NaCl, PEG, GA_3_) on germination rates and seedling vigor. Additionally, we investigated the impact of inhibitors (actinomycin D—inhibitor of transcription, cycloheximide—inhibitor of translation, hydroxyurea—inhibitor of DNA synthesis, cytochalasin—inhibitor of actin polymerization) on seed germination under stress conditions. Biochemical responses, including reactive oxygen species (ROS) levels and antioxidant enzyme activities, were analyzed to identify genotype-specific adaptations indicative of stress tolerance. Our results revealed significant variability in germination rates and seedling growth among the studied carrot experimental lines and commercial cultivars under salinity or drought stress Seed priming enhanced germination and seedling vigor by up to 35% under salinity stress and 28% under drought stress, with notable differences observed across the priming agents. The application of inhibitors highlighted the involvement of specific cellular processes in regulation of seed germination under stress. For instance, actinomycin D reduced germination by 40% under salinity stress. Biochemical analyses indicated genotype-specific responses, with variations in ROS levels and antioxidant enzyme activities such as superoxide dismutase and peroxidase. ROS levels increased by 50% under drought stress, whereas antioxidant enzyme activities varied substantially among genotypes. These findings underscored the importance of genotype-specific adaptations in conferring salinity or drought tolerance in carrot seedlings. Future research integrating omics approaches (e.g., transcriptomics, proteomics, metabolomics) will provide deeper insights into the molecular mechanisms that regulate stress tolerance, to aid in the development of more resilient carrot varieties suitable for cultivation under adverse environmental conditions.

## Introduction

Climate change poses substantial challenges to agriculture, necessitating the development of climate-smart crops that can withstand unpredictable weather patterns. Carrots (*Daucus carota* L.), a vital crop within the Apiaceae family, present unique challenges in terms of seed germination and seedling development due to their complex pollination biology [1–3] and outcrossing reproduction mode [4–6]. This often results in severe inbreeding depression if selfed, manifesting as low seed yield, loss of vigor, and fertility, or the expression of otherwise dormant recessive traits [1, 7–9]. Consequently, there is a pressing need to identify and develop carrot varieties that exhibit improved tolerance to abiotic stresses such as salinity and drought.

Previous studies have highlighted the variability in seed germination parameters among different carrot accessions, even under non-stress conditions [7, 10, 11]. This variability underscores the importance of understanding the genetic and physiological mechanisms underlying stress tolerance in carrots. These differences can be attributed to genetic diversity, seed quality, and environmental factors. Genetic factors include variations in the genetic makeup of different carrot varieties, which affect their physiological and biochemical responses to germination conditions. Environmental factors such as temperature, moisture, and soil conditions also play a significant role in influencing germination rates.

The use of seed priming and the application of various inhibitors have shown potential in analyzing seed germination and seedling vigor under stress conditions [12, 13]. But, the specific cellular processes and biochemical pathways involved in these responses remain largely unexplored. Seed priming is a pre-sowing treatment that involves the partial hydration of seeds to a point where germination processes begin but radicle emergence does not occur. This technique enhances seed performance by accelerating germination, improving seedling vigor, and increasing stress tolerance. Seed priming can be achieved through various methods, including hydropriming (soaking seeds in water), osmopriming (using osmotic solutions like PEG), and biopriming (using biological agents). The benefits of seed priming against abiotic stress conditions include enhanced germination and seed growth [14], improved stress tolerance [15], and increased yield and crop performance [16]. Seed priming has been explored as a technique to improve germination and seedling vigor in various crops, including carrots. Studies have shown that seed priming can enhance germination rates, reduce the time to germination, and improve seedling establishment under both optimal and stress conditions [12, 13]. For carrot seeds, priming with different agents has been reported to improve germination performance, particularly under abiotic stress conditions such as salinity and drought. The molecular mechanisms underlying the improvement of germination by seed priming involve several physiological and biochemical changes. Priming treatments can enhance the activation of metabolic processes, repair of cellular damage, and synthesis of proteins and enzymes necessary for germination. These processes include the activation of antioxidant defense systems, osmotic adjustment, and the regulation of hormonal pathways [15]. Priming can also lead to the accumulation of germination-promoting substances and the reduction of germination inhibitors.

Here, we hypothesized that the germination and early seedling growth of carrot seeds under salinity and drought stress are influenced by specific cellular processes and biochemical pathways. Specifically, we proposed that: (1) Seed priming with various agents (e.g., water, NaCl, PEG, GA_3_) would generally enhance germination rates and seedling vigor under stress conditions. (2) The application of inhibitors (actinomycin D, cycloheximide, hydroxyurea, cytochalasin) would reveal the involvement of specific cellular processes in regulation of seed germination under salinity or drought stress. (3) Genotype-specific biochemical responses, such as changes in reactive oxygen species (ROS) levels and antioxidant enzyme activities, would inform of differential tolerance to salinity and drought stress among carrot accessions. To test these hypotheses, our specific research goals were: (1) To evaluate the effects of seed priming with various agents on the germination and early seedling growth of a collection of carrot accessions’ seeds under salinity or drought stress. (2) To investigate the impact of specific cellular process inhibitors on seed germination under salinity or drought stress. (3) To analyze the biochemical responses, including ROS levels and antioxidant enzyme activities, in carrot seedlings under salinity or drought stress. (4) To identify genotype-specific adaptations that confer tolerance to salinity and drought stress in carrot seedlings. By completing these research goals, we aim to provide valuable insights into the mechanisms underlying salinity and drought tolerance in carrot, which will contribute to the development of more resilient carrot varieties suitable for cultivation under adverse environmental conditions.

## Materials and methods

### Plant materials

Seeds of carrot from the experimental male-fertile inbred lines (M1933; M1935; M1938; M1939; M1945) were developed in the National Institute of Horticulture, Department of Horticultural Crop Breeding, Skierniewice, Poland, 2008 through 2016. Seeds of the commercial cultivars “Jeanette F1”, “Koral”, “Perfekcja” were purchased from SPERLI, PNOS Ożarów Mazowiecki, and PlantiCo Zielonki Sp. z o.o., respectively. Care was taken to generate ample seed resources before onset of the experiment, so that all data was generated from the same seed lot, respectively. Seeds were not surface-sterilized, to imitate the real-life field conditions. Seeds were stored at 4 °C, to preserve seed viability and to ensure that the chilling requirement was met before germination assays [17].

### Seed germination assays

All germination assays were carried out in the plastic 10 × 10 cm square sterile plastic dishes Genoplast Biochemicals (Pruszków, Poland). The bottom dish was laid out with trimmed sterile Whatman #1 filter paper and soaked with 5 ml sterile solution–water as control, or solutions of tested agents or their combinations. Such prepared dish was used to put out seeds, with 4 × 25 seeds per tested accession placed in respective quadrant. Seeds were placed in the laminar flow hood, to prevent opportunistic microbial contamination. After covering the dish, it was placed in a plastic germination tray, laid out with sterile water-wetted paper towels and tightly covered with lid, to preserve high relative humidity (98% and higher). Germination was carried out at 22±1 °C, which is reported as the optimal temperature for carrot seed germination [3], in darkness–with periodic exposure to light for the purpose of documentation and counting the germinated seeds.

Seeds were inspected for germinated radicle twice daily, at 8am and 4pm. Seed was counted as germinated if a visible radicle equal half of the seed length was observed protruding from the seed. Score of germinated seeds were tabulated, and a given seed was marked as germinated on the lid of the dish using a permanent marker, to prevent its repetitive counting.

### Screens for salt and drought tolerance

Seeds of carrot experimental lines and commercial cultivars were screened for tolerance to salt stress, by exposure to 50, 100, and 150 mM of NaCl, or their respective equivalents for KCl or NPK nutrient (Agrolution Special 324; Everris, Geldermalsen, The Netherlands), based on the electric conductivity [μS]. Similarly, the increasing concentrations of PEG 8000 (5, 10, 15%, and 20%; v/v) were tested to imitate the drought stress [18–20], but the initial screens showed that all PEG concentrations except the lowest ones resulted in severe germination inhibition. The optimal screening concentrations of NaCl and PEG established were used for subsequent experiments.

### Screens for germination priming

Improvements of germination were assessed by priming the seeds of line M1933, deemed the sensitivity standard, in each of the following agents: water; 50 mM NaCl; 5% v/v PEG 8000; and 100 μM GA3 (Duchefa Poland). The concentrations of these priming agents were selected based on previous studies demonstrating their effectiveness in enhancing seed germination and seedling vigor under stress conditions [14, 21, 22], as well as based on the data generated in this study. Seeds were primed at 22±1 °C for 12 h, after which time they were dried in a desiccator at 22±1 °C for 48 h. Subsequently, each seed lot was subjected to germination assays in water, NaCl, or PEG solutions to evaluate the priming effects. This approach ensured that the priming effects were temporally separated from the inclusion effect of the priming agents. Germination assays were carried out as described above.

### Screens for salt, drought tolerance with metabolic inhibitors

To assess the major mechanisms governing salt or drought tolerance, the accessions assessed as sensitive and tolerant were further tested. We did this by comparing germination in water to that in NaCl or PEG, to that with either stress agent but independently amended with 100 μM of each of the following inhibitors: actinomycin D (inhibitor of transcription; AppliChem, A1489); cycloheximide (inhibitor of translation; Amresco, 94271); hydroxyurea (inhibitor of DNA synthesis; Sigma-Aldrich, H8627); and cytochalasin (inhibitor of actin polymerization; Cayman Biochemical Company, 1-800-364-9897). The choice of inhibitor concentrations was based on previous studies demonstrating their effectiveness in inhibiting specific cellular processes in plant systems [12, 13, 23, 24]. Germination assays were carried out as described above.

### Seedlings’ growth dynamics

One-week old seedlings of the accessions deemed salt-sensitive (M1933) and salt-tolerant (M1945) germinated in water or NaCl were subjected to the following assay. Seedlings were placed in new square 10 cm Petri dishes laid with Whatman paper and moistened with water or salt-imposing solution, to assess their ability to adjust to stress imposition or retraction (i.e., combinations of water germinated–water grown; water germinated–NaCl grown; NaCl germinated–water grown; and NaCl germinated–NaCl grown). Seedlings were 7 days old at the start of the experiment. Root and shoot lengths were measured at 7 days after transfer to such new dishes, transferred to another set of new Petri dishes, and measured again at 14 days after the initial transfer, transferred to new dishes, and measured again at 21 days after the initial transfer. Growth dynamics were plotted from the root, shoot lengths, and the Root:Shoot length ratios at 7, 14, and 21 days after the initial transfer, in R v.4.3.2 using the package ggplot2 v.3.5.1 [25].

### Assessment of biochemical responses and enzymatic activities

#### Plant material and growth conditions

Seven-day-old carrot seedlings were grown under respective conditions (control, salinity, and drought stress). Seedlings were harvested, weighted, and immediately processed for biochemical assays. Tissue was homogenized in a 2:1 (v/w) ratio with the respective solvent system, precooled to 4 °C, similar to our biochemical investigations of other systems [26–28].

#### Assessment of reactive oxygen species (ROS) generation (O_2_^−^)

The generation of ROS species, specifically superoxide anion (O_2_^−^), was assessed following the published methods [29–32]. Methanolic extracts were prepared from the homogenized tissue and assayed at A530 for O_2_^−^. The absorbance readout for O_2_^−^ assays was recalculated per mg of fresh tissue.

### Enzyme activity assays

The activities of polyphenol oxidase (PPO), peroxidase (POX), superoxide dismutase (SOD), and catalase (CAT) were analyzed using methods published previously [27, 32, 33]. Buffer-extracted samples were assayed for protein content using the BCA kit (Novazym, Poznań, Poland). Enzyme assays were conducted in microtiter plates, with absorbance readouts recorded every minute for 3 to 5 minutes using an Epoch2T reader (BioTek Instruments, Inc., Bad Friedrichshall, Germany). Enzyme activities were calculated for PPO as the change in absorbance per minute per mg of protein, for POX, SOD, CAT as metabolized substrate per minute per mg of protein.

#### Catalase (CAT) activity

CAT activity was determined by measuring the decrease in absorbance of H_2_O_2_ at 240 nm for 3 minutes using a spectrophotometer (Shimadzu AA-1208, Kyoto, Japan). The reaction mixture contained 50 mM KH2PO4 (pH 7), 13 mM H_2_O_2_, and 30 μL enzyme extract. One unit of CAT activity was defined as the amount of enzyme catalyzing the decomposition of 1 μmol H_2_O_2_ per minute, calculated using the extinction coefficient (0.036 cm^2^/μmol) for H_2_O_2_ at 240 nm [26, 34].

#### Superoxide dismutase (SOD) activity

Total SOD activity was determined by monitoring the inhibition of the reduction of p-nitro-blue tetrazolium chloride (NBT) [26, 35]. The reaction mixture contained 50 mM phosphate buffer (pH 7.8), 0.1 mM EDTA, 63 μM NBT, 50 μM riboflavin, 13 mM methionine, and 50 μL extract. The reaction was initiated by illumination with a 22 W fluorescent lamp, and the reduction of NBT was followed by reading the absorbance at 560 nm for 10 minutes. One unit of SOD was defined as the amount of enzyme producing a 50% inhibition of NBT reduction.

#### Polyphenol oxidase (PPO) activity

PPO activity was measured using the methods described previously [26, 36]. Root extracts (200 μL) were mixed with 700 μL of homogenization buffer. The rate of increase in absorbance at 420 nm was measured for 1 minute after the addition of 100 μL 0.2 M catechol. Mushroom PPO (Sigma Aldrich, Warsaw, Poland) was used as the standard, and results were expressed as μg PPO per mg protein.

#### Peroxidase (POX) activity

POX activity was determined following the previously published methodology [26, 36]. Root tissue extracts were diluted 10-fold with homogenization buffer. One hundred μL of the diluted extracts were added to 792 μL of 5 mM sodium phosphate buffer (pH 6.0), mixed with 7.5 μL guaiacol (60 mM), and the reaction was initiated by adding 100 μL 0.6 M H_2_O_2_. The initial rate of increase in absorbance at 470 nm was measured over 1 minute. Horseradish peroxidase (ICN, Biochemicals, Costa Mesa, CA, USA) was used as the standard, and POX activity was expressed as units of POX per mg protein.

### Lignin deposition

Levels of lignin deposition were assessed using published spectrophotometric methods [37–39]. Methanolic extracts and pellets were used, with technical lignins (Sigma Aldrich) serving as the respective standards.

### Statistical analyses

Germination data were analyzed using GerminatoR v.2.01 [40], with the input of the germinated seed counts tabulated using MS Excel. Each experiment used at least 4 replicates of 25 seeds per tested accession per condition, with experiments repeated at least twice. GerminatoR calculates the high-throughput scoring and curve fitting of seed germination [40]. Comparisons of Area Under Curve were done at maximum germination time per each analyzed dataset. Biochemical analyses were performed in at least triplicate technical repetitions, with at least duplicate biological repetitions. Factorial ANOVA with post-hoc Fisher’s Honestly Significant Difference at α = 0.05 was run in R v.4.40 [41] as implemented in package agricolae v.4.4.1 [42].

## Results

### Germination screens—Effects of salinity and drought

In this study, we aimed to evaluate the germination performance of various carrot accessions under salinity and drought stress conditions (S1 Fig). Salinity and drought tolerance screens in the carrot germplasm were based on testing a large pool of seeds: Germination tests used in total 6,025 seeds (NaCl; screen optimization) or 2,425 seeds (KCl; NPK; PEG, respectively). Priming assays used in total 1,675 seeds; inhibitors assays used in total 7,575 seeds. These estimates are conservative, as several assays’ data were not included in the calculations due to high initial concentrations used that resulted in no germination. Thus, the following observations are based on robust germination data.

**Fig 1:**
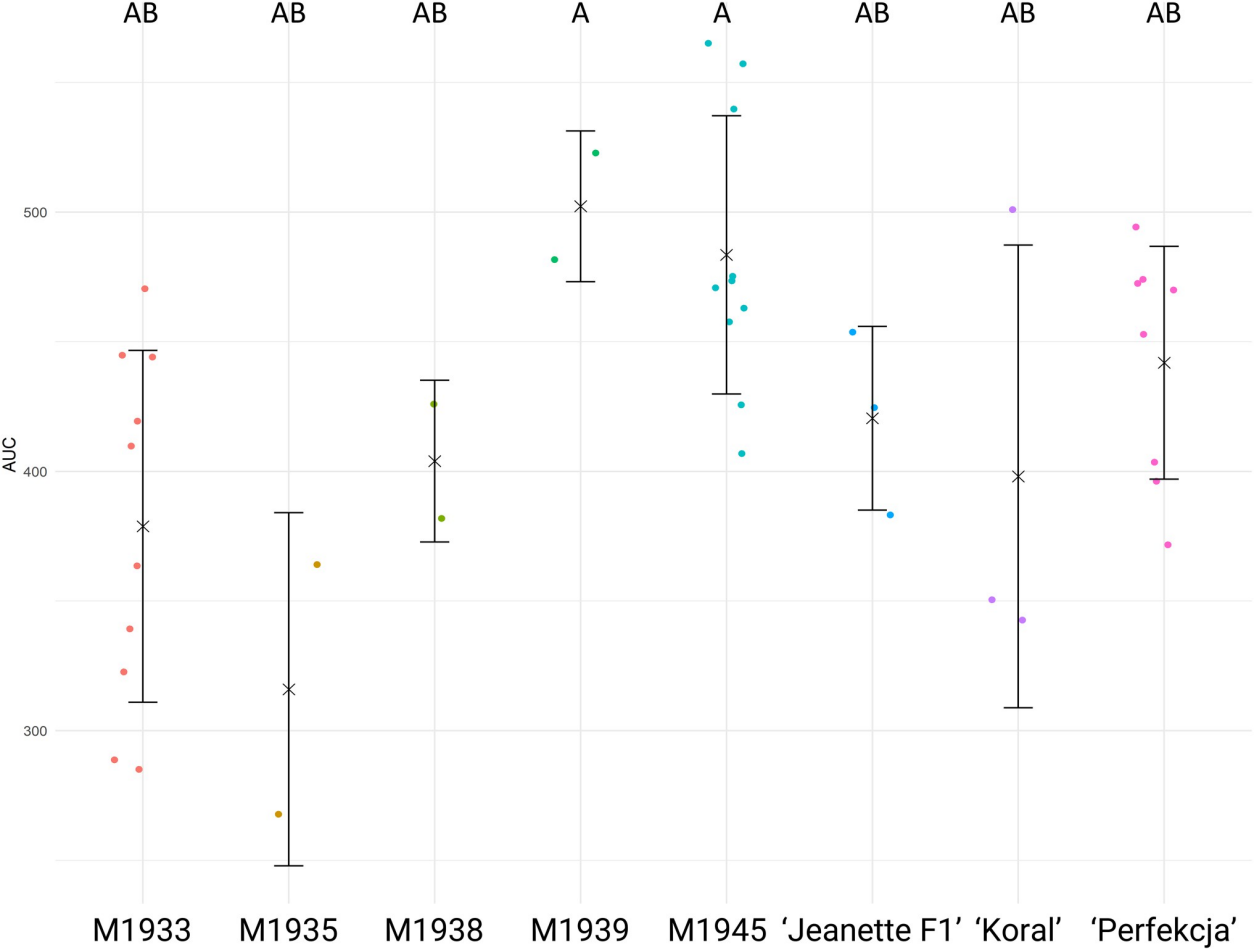
Results of carrot seed germination in water. Raw data for Area Under Curve (AUC, vertical axis) are shown for each tested accession (horizontal axis), accompanied by means (× symbols) and standard deviations (whiskers), respectively. Each dot corresponds to the AUC score calculated based on germination rates of 25 seeds, calculated in GerminatoR. After single factorial ANOVA, a test for Tukey’s Honestly Significant Differences was carried out at α = 0.05; the results are indicated by lettering–groups with the same letter code are not significantly different. The critical value of the Studentized Range for α = 0.05 was determined to be 4.58106.

The germination of various tested carrot seeds in water was analyzed to assess their performance under such defined baseline conditions (Fig 1). Significant differences in germination rates among the tested carrot accessions were detected. Carrots lines M1939 and M1945 exhibited comparably higher germination rates than other tested accessions. The ANOVA results revealed a statistically significant effect of carrot genotype on germination performance (F_(7, 32)_ = 4.153, p = 0.00237).

**Fig 2:**
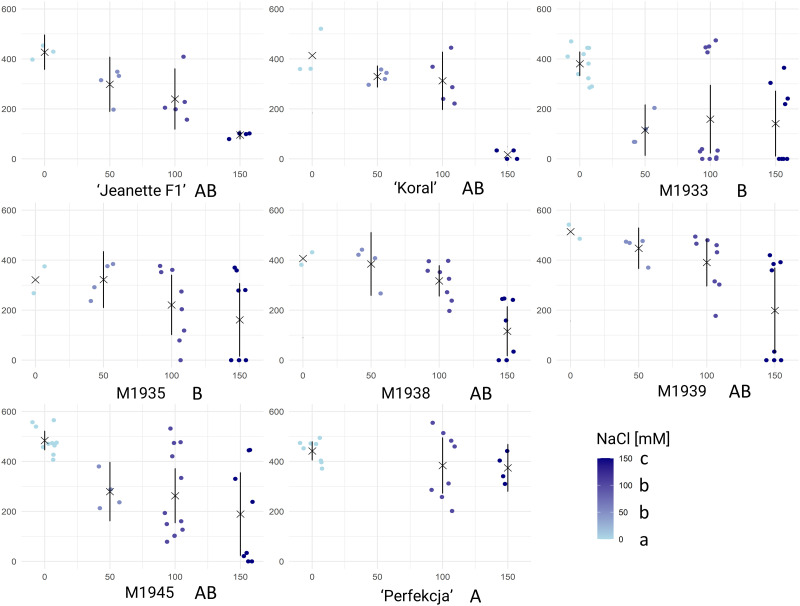
Results of carrot seed germination in varying concentrations of NaCl. (indicated in legend, bottom-right). Raw data for Area Under Curve (AUC, vertical axis) are shown for each tested accession and NaCl concentration (horizontal axis), respectively, accompanied by means (× symbols) and standard deviations (whiskers), respectively. Each dot corresponds to the AUC score calculated based on germination rates of 25 seeds, calculated in GerminatoR. After single factorial ANOVA, a test for Tukey’s Honestly Significant Differences was carried out at α = 0.05 independently for carrot genotypes and NaCl concentration; the results are indicated by lettering (capital lettering for accessions; small lettering for NaCl concentration); groups with the same letter code are not significantly different. The critical value of the Studentized Range for α = 0.05 was determined to be 4.337872 (carrot accession) and 3.66695 (NaCl concentration), respectively.

The germination of different tested carrot seeds under various NaCl concentrations was investigated to assess their tolerance to salt stress. The ANOVA results (S1 Table) indicated that the germination of carrot seeds was significantly influenced by both the carrot genotype and the NaCl concentration. The cultivar "Perfekcja" exhibited the highest germination rate, whereas M1933 and M1935 showed relatively lower germination rates (Fig 2). Additionally, the germination rates decreased with increasing NaCl concentration, indicating the negative impact of salt stress on carrot seed germination. Significant effects were seen of both carrot genotype (F_(7, 170)_ = 5.567, p = 8.67e-06) and NaCl concentration (F_(1, 170)_ = 92.190, p < 2e-16) on the germination performance. Additionally, there was no significant interaction between carrot genotype and NaCl concentration (F_(7, 170)_ = 1.519, p = 0.164).

**Fig 3:**
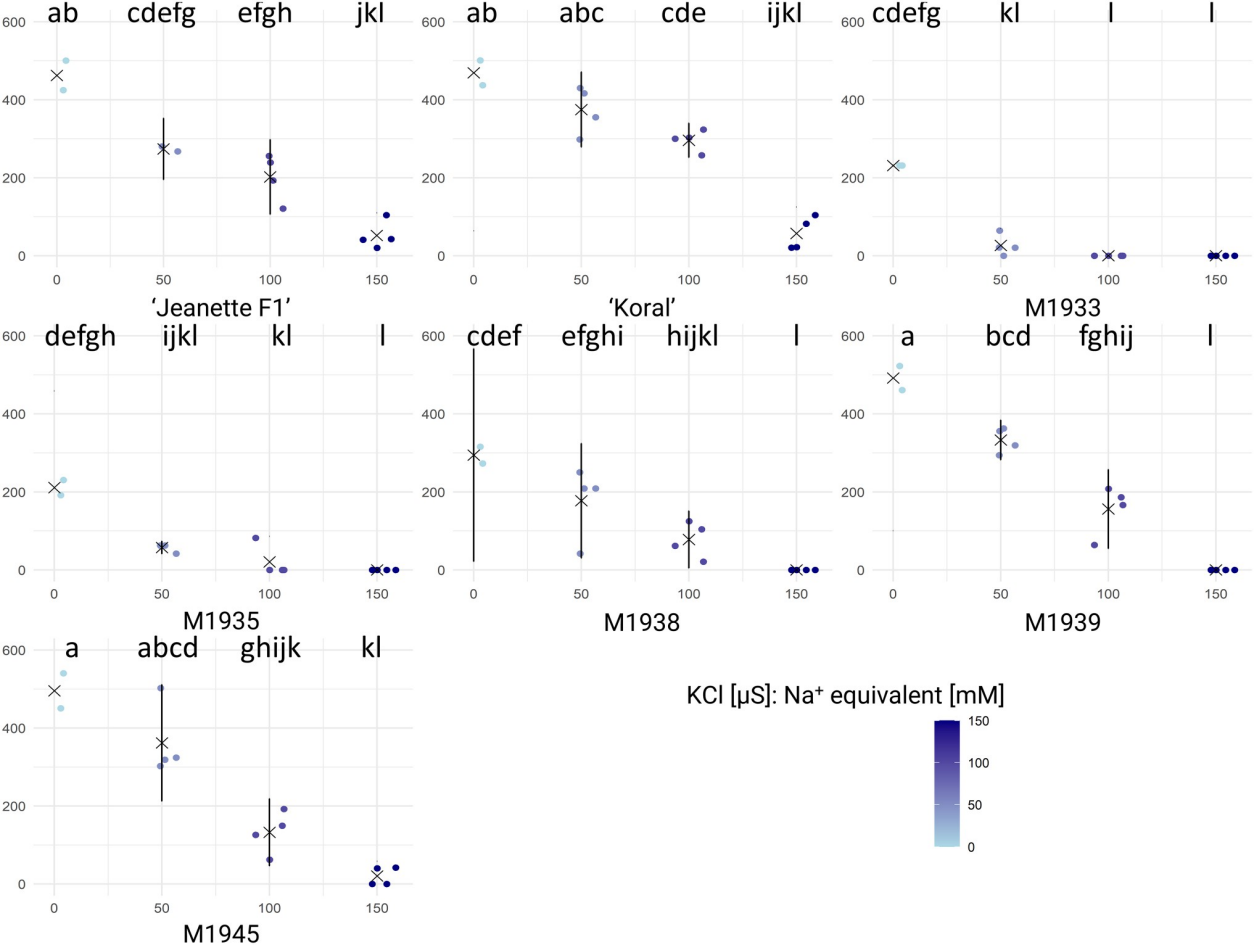
Results of carrot seed germination in varying concentrations of KCl. (indicated in legend, bottom-right). Raw data for Area Under Curve (AUC, vertical axis) are shown for each tested accession and KCl concentration (horizontal axis), respectively, accompanied by means (× symbols) and standard deviations (whiskers), respectively. Each dot corresponds to the AUC score calculated based on germination rates of 25 seeds, calculated in GerminatoR. After 2-way ANOVA, a test for Tukey’s Honestly Significant Differences was carried out at α = 0.05 for interaction between carrot accessions and KCl concentration; the results are indicated by lettering; groups with the same letter code are not significantly different. The critical value of the Studentized Range for α = 0.05 was determined to be 5.481419.

The germination of different tested carrot seeds under various KCl concentrations was examined to assess their tolerance to such induced stress at electric conductance levels equal to NaCl, respectively. Germination of carrot seeds was significantly influenced by both the carrot genotype and the KCl concentration. Carrot accessions exhibited variable levels of tolerance to KCl-induced stress, with some accessions maintaining higher germination rates compared to others across different KCl concentrations (Fig 3). Commercial cultivars ‘Jeanette F1’ and ‘Koral’ performed comparably the best, with germination rates exceeding those for other tested carrot genotypes. But, considering the effect of 50 mM NaCl, they lacked differences from M1939 or M1945; whereas at 100 mM NaCl, neither M1939 nor M1945 performed better than ‘Jeannette F1’. The ANOVA results (S1 Table) reveal significant effects of carrot accession (F_(6, 82)_ = 40.58, p < 2e-16), KCl concentration (F_(1, 82)_ = 491.32, p < 2e-16), and their interaction (F_(6, 82)_ = 11.21, p = 4.22e-09) on germination performance.

**Fig 4:**
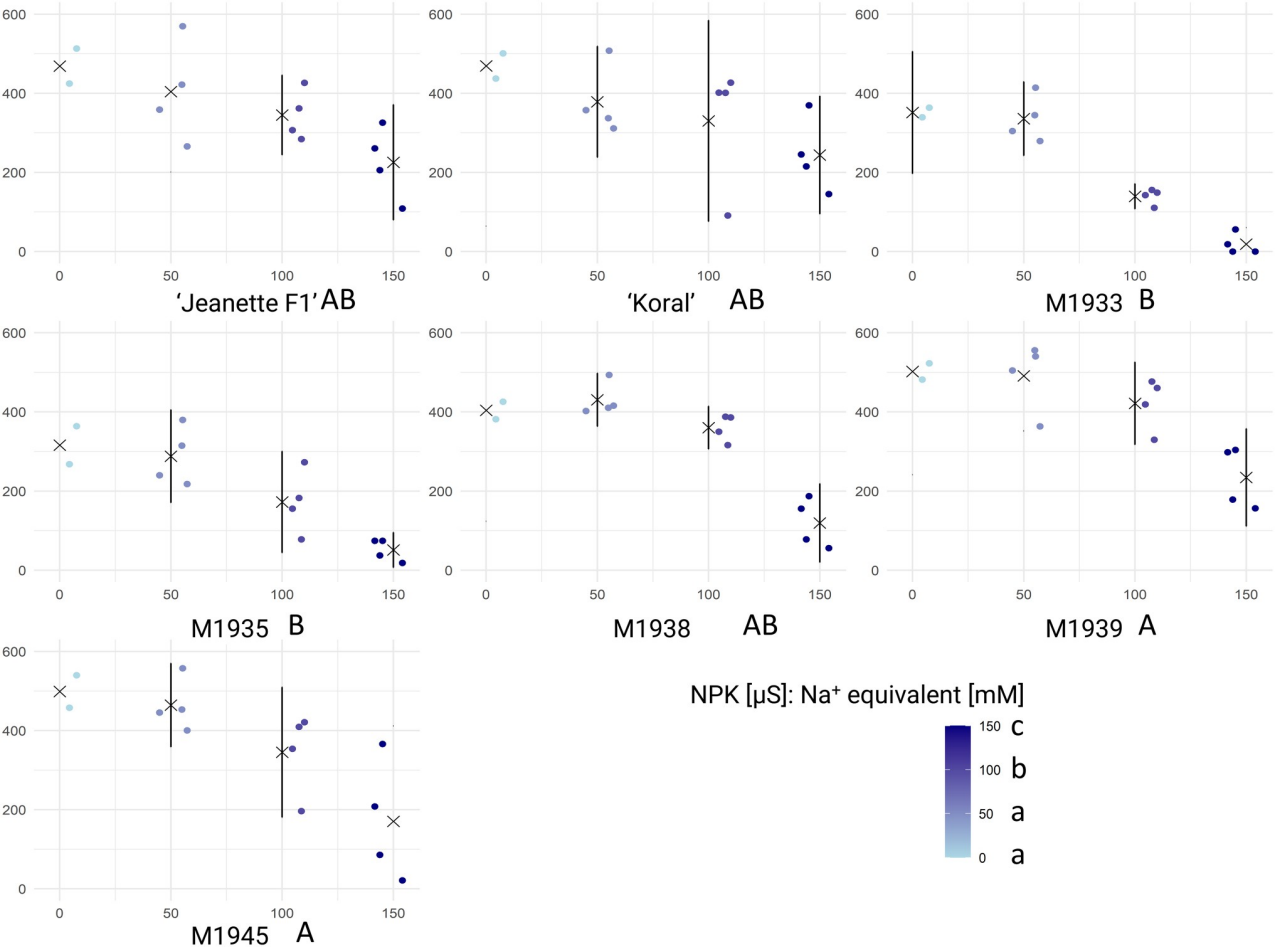
Results of carrot seed germination in varying concentrations of NPK. (indicated in legend, bottom-right). Raw data for Area Under Curve (AUC, vertical axis) are shown for each tested accession and NPK concentration (horizontal axis), accompanied by means (× symbols) and standard deviations, respectively. Each dot corresponds to the AUC score calculated based on germination rates of 25 seeds, calculated in GerminatoR. After single factorial ANOVA, a test for Tukey’s Honestly Significant Differences was carried out at α = 0.05 independently for carrot accessions and NPK concentration; the results are indicated by lettering (capital lettering for genotypes; small lettering for NPK concentration); groups with the same letter code are not significantly different. The critical value of the Studentized Range for α = 0.05 was determined to be 155.0371 (carrot accession) and 3.699002 (NPK concentration), respectively.

Inhibition of germination can result from local over-fertilization. Germination of various tested carrot seeds under various NPK concentrations, simulating over-fertilization, was analyzed to understand how the excessive nutrient levels affect germination performance. These findings suggest differential responses of carrot accessions to varying NPK concentrations, with lines M1939 and M1945 exhibiting higher tolerance to over-fertilization conditions compared to other tested accessions (Fig 4). ANOVA results (S1 Table) indicate significant effects of carrot genotype (F_(6, 84)_ = 12.94, p = 2.64e-10) and NPK concentration (F_(1, 84)_ = 145.03, p < 2e-16) on germination performance. The interaction between carrot genotype and NPK concentration was not significant (F_(6, 84)_ = 0.83, p = 0.55).

**Fig 5:**
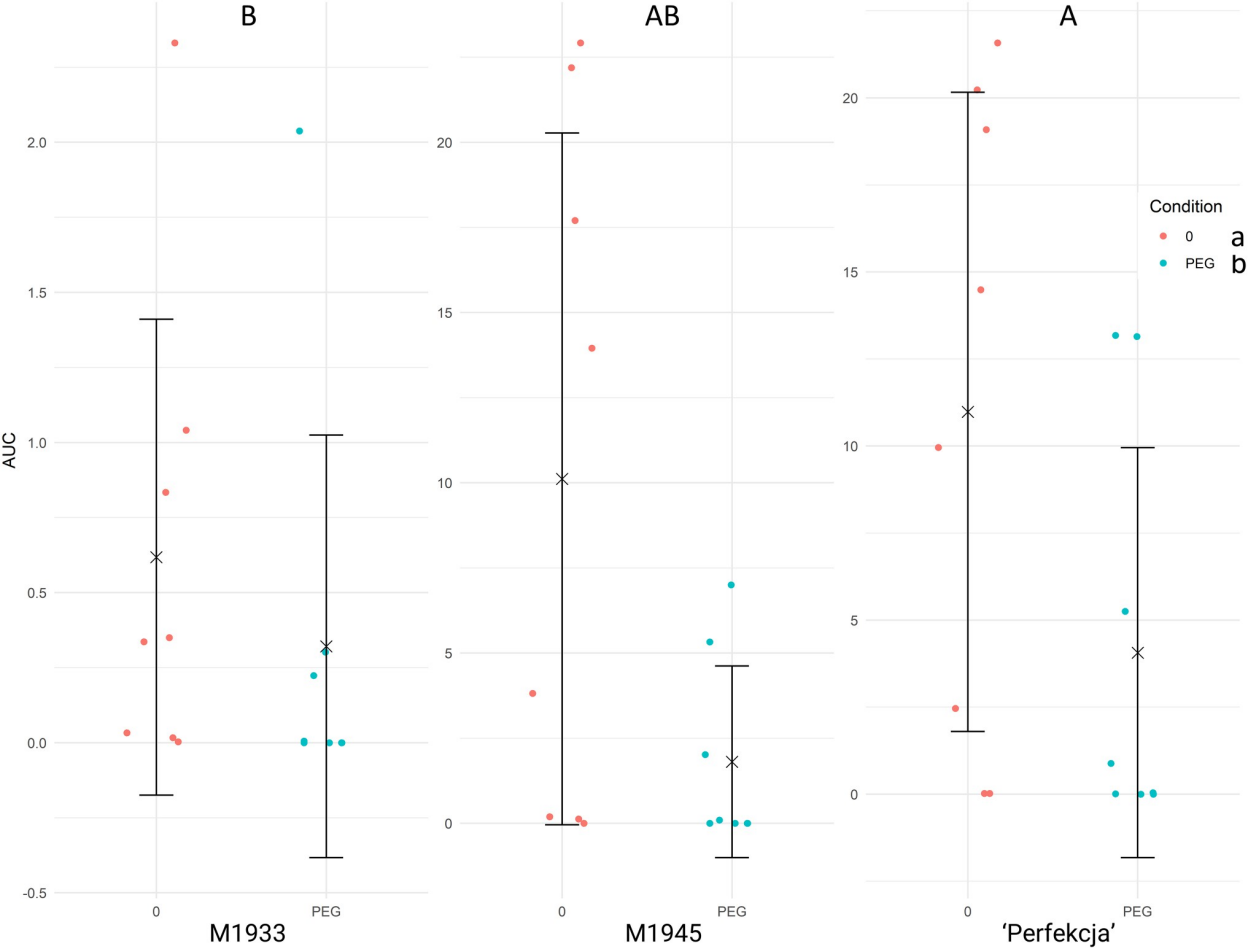
Results of carrot seed (M1933, M1945, ‘Perfekcja’) germination under drought stress imposed by PEG. (indicated in legend, right). Raw data for Area Under Curve (AUC, vertical axis) are shown for each tested accession under water (0) and 5% w/v PEG treatments (horizontal axis), respectively, accompanied by means (× symbols) and standard deviations (whiskers), respectively. Each dot corresponds to the AUC score calculated based on germination rates of 25 seeds, calculated in GerminatoR. After single factorial ANOVA, a test for Tukey’s Honestly Significant Differences was carried out at α = 0.05 independently for carrot accessions and test conditions (water vs. PEG); the results are indicated by lettering (capital lettering for genotypes; small lettering for condition); groups with the same letter code are not significantly different. The critical value of the Studentized Range for α = 0.05 was determined to be 3.424983 (carrot accession) and 2.845031 (PEG concentration), respectively.

Germination of different tested carrot seeds under polyethylene glycol (PEG) stress, that simulated drought conditions, was analyzed to understand how that stress affects germination performance. Compared with salinity, carrot seeds showed a higher sensitivity towards drought. The initial screens tested the PEG 8000 concentrations at 10, 15, and 20%, w/v but yielded no germination of any of the tested carrot accessions. As such, the next round of screens used 5% w/v of PEG 8000, which discriminated among the tested carrot germplasm (Fig 5) and was used for subsequent experimentation. At that drought level, the experimental line M1945 and commercial cultivar ‘Perfekcja’ stood out as particularly drought-tolerant plant materials, and therefore were used as tolerance standards henceforth. In contrast, M1933 performed poorly under such imposed drought or in water and served as the sensitivity standard in the following experiments. The ANOVA results (S1 Table) indicate significant effects of the carrot accession (F_(2, 43)_ = 6.111, p = 0.00462) and PEG presence (F_(1, 43)_ = 8.424, p = 0.00582) on germination performance. The interaction between carrot genotype and PEG concentration was not significant (F_(2, 43)_ = 2.002, p = 0.14739).

### Effects of metabolic inhibitors on carrot seed germination

**Fig 6:**
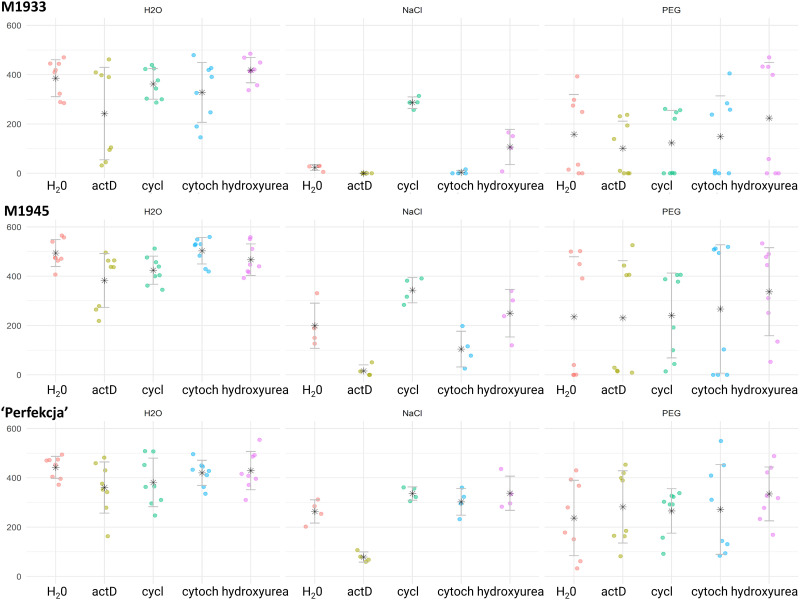
Results of carrot seed germination under drought or NaCl stresses and impacts of metabolic inhibitors. Carrot genotypes tested (M1933, M1945, ‘Perfekcja’) are indicated. Raw data for Area Under Curve (AUC, vertical axis) are shown for each tested accession accompanied (horizontal axis) by means (* symbols) and standard deviations, respectively, under water (H_2_O), NaCl, and PEG treatments, respectively. Each dot corresponds to the AUC score calculated based on germination rates of 25 seeds, calculated in GerminatoR. Each condition was also tested for the effects of four inhibitors: actinomycin D (actD); cycloheximide (cycl); cytochalasin (cytoch), and hydroxyurea, as compared to germination in water (H_2_O). Post-ANOVA Tukey groupings are omitted from the figure for clarity of presentation–see S1 Table).

Seed germination can be affected at several important developmental processes, such as transcription, translation, DNA synthesis, or cell division. We tested the inhibitors specific to each of these processes (actinomycin D, cycloheximide, cytochalasin, hydroxyurea, respectively), to assess which of them and to what extent regulate the germination of carrot seeds under salinity or drought, compared to germination in water (Fig 6). The results demonstrate that both germination conditions and inhibitors significantly influenced seed germination rates across the tested carrot genotypes (M1933, M1945, ‘Perfekcja’). Importantly, no major germination decreases in water were noted for any of the assayed carrot accessions for any of the four tested inhibitors. This analysis highlighted the unexpected effect of germination activation under salinity in all tested genotypes exerted by cycloheximide and, to a lesser degree, by hydroxyurea. Germination in both salt-tolerant genotypes M1945 and ‘Perfekcja’ was severely inhibited by actinomycin D. No specific reactions were observed under PEG, with a minor decrease in germination under cycloheximide treatment. The ANOVA results (S1 Table) indicate significant effects of genotype (F_(2, 255)_ = 23.489, p = 4.33e-10), condition (F_(2, 255)_ = 80.953, p < 2e-16), inhibitor (F_(4, 255)_ = 6.954, p = 2.50e-05), and the interaction between genotype and condition (F_(4, 255)_ = 2.204, p = 0.0690), and condition and inhibitor (F_(8, 255)_ = 3.363, p = 0.0011). But, the interactions between accession and inhibitor (F_(8, 255)_ = 0.290, p = 0.9688) and genotype, condition, and inhibitor (F_(16, 255)_ = 0.590, p = 0.8906) were not statistically significant. The critical value of the Studentized Range for α = 0.05 was determined to be 3.331215 (carrot genotype; Minimum Significant Difference: 55.51681); 3.331215 (germination condition–H_2_O, NaCl, PEG); 3.881627 (inhibitor, if any; Minimum Significant Difference: 85.41221); 4.419856 (interaction genotype × condition); 4.838706 (interaction condition × inhibitor), respectively.

### Effect of seed priming on carrot seed germination

**Fig 7:**
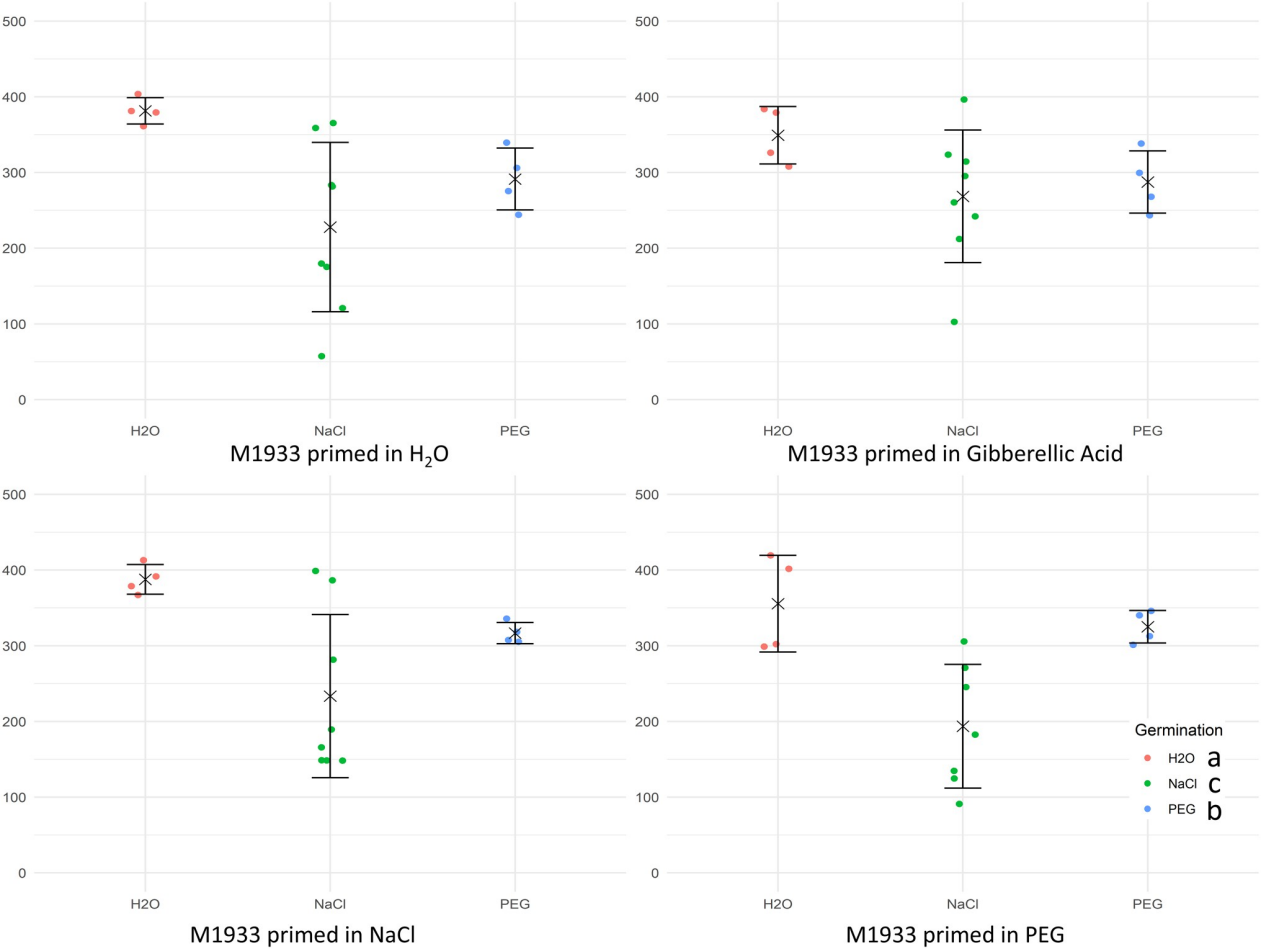
Results of carrot seed germination (M1933; sensitivity standard) after priming using several compounds. (water, 50 mM NaCl; 5% v/v PEG 8000; or 100 μM GA_3_), and germination under various conditions (water, 50 mM NaCl, or 5% v/v PEG 8000). Raw data for Area Under Curve (AUC, vertical axis) are shown for each tested accession (horizontal axis) accompanied by means (× symbols) and standard deviations (whiskers), respectively. Each dot corresponds to the AUC score calculated based on germination rates of 25 seeds, calculated in GerminatoR. Post-ANOVA Tukey groupings are indicated per each germination condition in the legend (bottom-right).

Seed priming was tested to assess the potential for improvement of the generally poor seed germination in Apiaceae, as compared to other vegetable species. Pre-treatments with water, gibberellic acid, NaCl, or PEG were applied and their effects on germination of M1933, the sensitivity standard, under various conditions (H_2_O, NaCl, and PEG) were assessed (Fig 7). The choice of priming substance did not significantly affect germination outcomes under the tested conditions. But, significant differences in the germination rates were observed under the tested conditions, with H_2_O treatment (no stress) resulting in the highest germination rates. The ANOVA results (S1 Table) reveal that the main effects of priming (F_(3, 51_) = 0.280, p = 0.839) and the interaction between priming and germination conditions (F_(6, 51)_ = 0.671, p = 0.673) were not statistically significant. Only the main effect of germination conditions was highly significant (F_(2, 51)_ = 17.952, p = 1.25e-06). The critical value of the Studentized Range for α = 0.05 was determined to be 3.398661.

### Seedling growth dynamics

**Fig 8:**
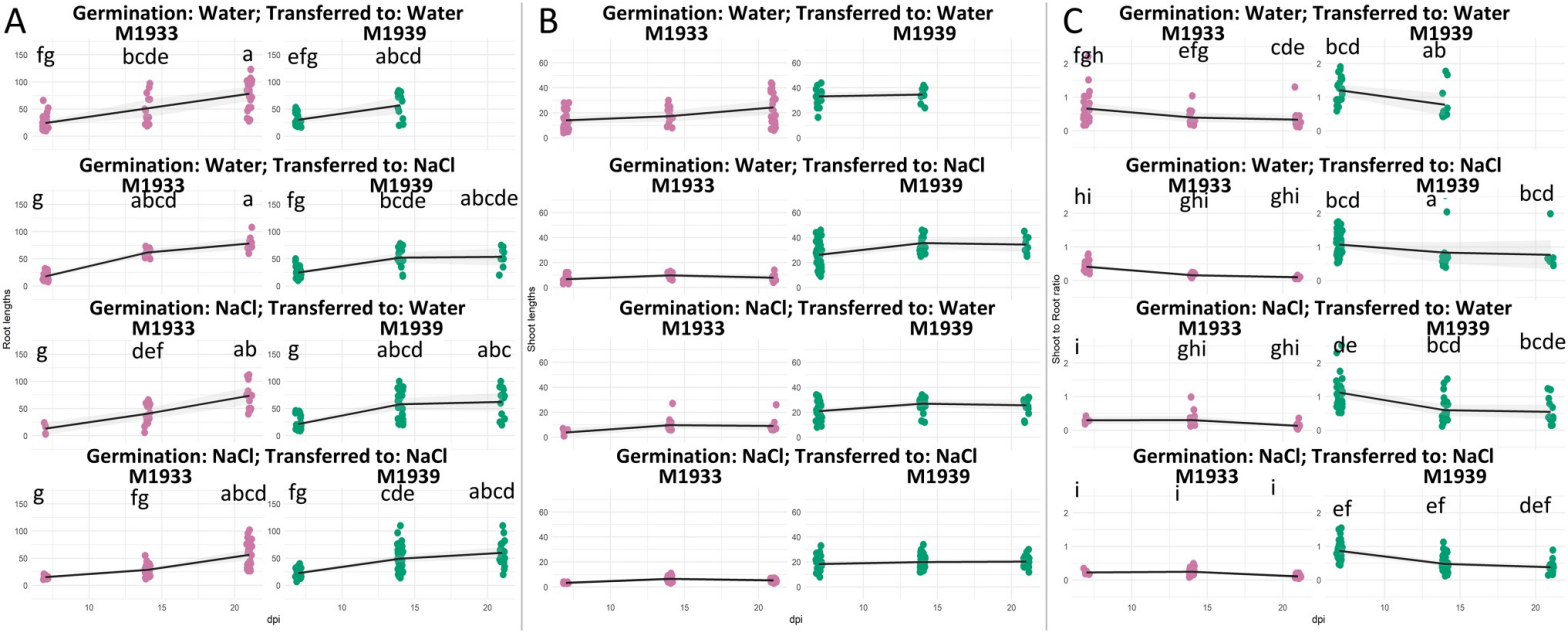
Dynamics of carrot seedling growth. Graphs show (A) root length [mm]; (B) shoot length [mm]; (C) Root:Shoot lengths ratio (vertical axes). Each dot corresponds to the growth score noted at a given time and for each indicated carrot genotype (horizontal axis). The 95% confidence intervals are indicated by grey shading. Seeds of M1933 (NaCl sensitivity standard) and M1945 (tolerance standard) were germinated for 7 days under indicated conditions, and then transferred at 14 and 21 days. Root and shoot lengths were measured each 7 days. Statistically different groups in A and C for interaction of genotype × time × growth conditions are indicated; groups for B showed significance for all three double interactions only and are presented in S1 Table. Note: No seedlings were recovered for M1939 control (germinated in water; transferred to water) at 21 dpi.

Analyses of seedling growth dynamics can help estimate the extent of possible losses upon salinity or drought imposition, or–contrastingly–potential avoidance of losses if the stress subsides (S2 Fig). Both individual factors (Accession, Condition, DPI) and their 2-way and 3-way interactions significantly influence root length in carrot seedlings under variable salinity treatments (Fig 8A; S1 Table). Post hoc analyses further elucidated specific differences between carrot accessions and germination conditions and indicated that the sensitive line M1933 developed the longest roots, unless germinated and grown in NaCl.

The results of the single factorial and 2-way ANOVA suggest that both individual factors (Accession, Condition, DPI) and their interactions significantly influence shoot length in carrot seedlings (Fig 8B). The main effect of genotype (M1933 vs. M1945) determined the interaction of genotype and DPI. Similarly, the genotype had a strong effect on separation of interactions with growth conditions, with the sensitive line M1933 performing comparably worse. Finally, in the interaction of growth conditions with DPI, germination in NaCl showed strong effects on the shoot length.

The Root:Shoot lengths ratio of carrot seedlings was significantly influenced by the type of carrot accession, germination conditions, and the duration of growth post imbibition. Interactions between these factors shaped the root-to-shoot ratio, highlighting the complexity of factors affecting plant growth and development in carrot seedlings. The outcomes of the sensitive line M1933 were comparably the worst, regardless of the other factors.

### Biochemical basis of salinity and drought tolerance

**Fig 9:**
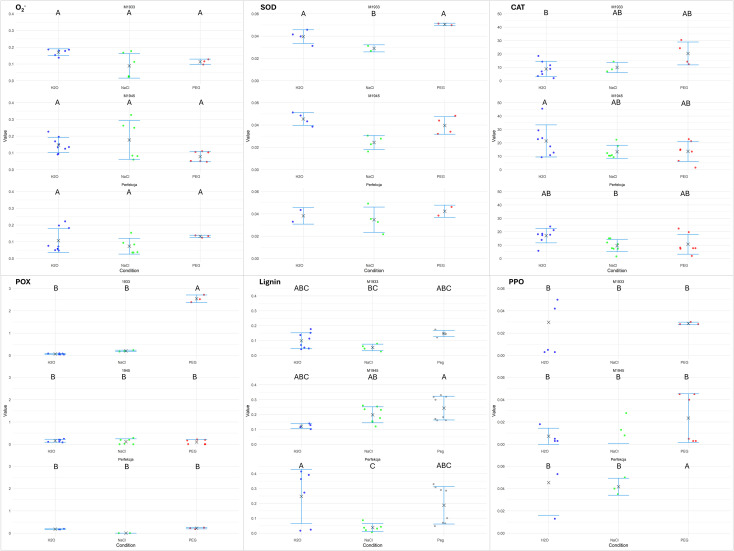
Analyses of biochemical parameters of carrot seedlings. (M1933, M1945, ‘Perfekcja’) germinating under water (H_2_O) or under imposed salinity (NaCl) or drought (PEG). Shown are raw data for each assay, recalculated per mg dry weight (and 1 min of reaction, wherever applicable) alongside the means (× symbols) and standard errors (whiskers). Each dot corresponds to the result of a single biochemical assessment, as an average of three technical tests. Vertical axes–values for a given biochemical parameter tested; horizontal axis–carrot seedlings and testing conditions.

Analyses of ROS levels, ROS detoxifying enzymes (SOD, CAT, and POX), and protective metabolites (lignins, PPO) in the germinating carrot seedlings showed their involvement during germination under salinity and drought stress (Fig 9). Some of the observed biochemical responses are common across accessions, such as the decrease in SOD activity under salinity stress, whereas others are specific to certain genotypes or stress conditions, which indicates genotype-specific adaptations.

Levels of O_2_^-^ showed significant differences only in the ‘Perfekcja’ seedlings under drought stress, suggesting a specific response to drought in this accession. The O_2_^-^ ANOVA (S1 Table) revealed that the interaction between genotype (genotype) and germination conditions (F_(4, 45)_ = 0.011030, p = 0.0304) were statistically significant with the critical value of the Studentized Range determined at 4.606285.

The SOD activity that detoxifies that ROS species was significantly decreased in all tested carrot accessions only under salinity stress, which indicated a common response to salinity across the accessions. The SOD ANOVA revealed that only the germination conditions (F_(2, 19)_ = 10.261, p = 0.000951) were statistically significant with the critical value of the Studentized Range determined at 3.522566.

The activity of CAT was significantly increased only in the tolerant M1945 germinating in water, and significantly decreased in the sensitive M1933 in water as well as in the tolerant ‘Perfekcja’ under salinity. The CAT ANOVA revealed that the interaction between accession and germination conditions (F_(4, 51)_ = 3.344, p = 0.0166) were statistically significant with the critical value of the Studentized Range determined at 4.579915.

The activity of POX was significantly increased only in M1933 under drought. The POX ANOVA revealed that the interaction between genotype (genotype) and germination conditions (F_(4, 29)_ = 2.3992, p = <2e-16) were statistically significant with the critical value of the Studentized Range determined at 4.731843.

Levels of lignin were significantly decreased in ‘Perfekcja’ under salinity, indicating a potential vulnerability to salinity-induced damage, whereas the activity of PPO involved in lignin metabolism was significantly higher only in ‘Perfekcja’ under drought. The lignin ANOVA revealed that the interaction between genotype (genotype) and germination conditions (F_(4, 47)_ = 3.789, p = 0.00946) were statistically significant with the critical value of the Studentized Range determined at 4.596731. The PPO ANOVA revealed that the interaction between accession and germination conditions (F_(4, 29)_ = 2.3992, p = <2e-16) were statistically significant with the critical value of the Studentized Range determined at 4.731843.

## Discussion

Ongoing climate change necessitates the search for climate-smart crops, better suited to unpredictable weather patterns than the currently used germplasm. With complex pollination biology, seeds of the Apiaceae crops present a unique challenge with regards to germination assessments. Generally outcrossing with severe inbreeding depression if selfed, carrots rely on insect for cross-pollination and on specific environmental conditions to realize their reproduction potential [1, 2, 5, 6]. The random naturally occurring self-pollination, or one intended for cultivar uniformity alignment, results in severe inbreeding depression, which manifests in low seed yield, loss of vigor and fertility, or expression of otherwise dormant recessive traits [1, 3, 4, 6, 7].

Open-pollinated varieties/cultivars tend to suffer from less uniformity in the population, but F1 hybrids tend to be comparatively more uniform [3, 4, 6–8]. Unless mass-grown for seed purposes using plants of homogenous genotypes, for instance coming from micropropagation or tissue culture [10, 11, 43, 44], high variability in seed germination parameters is expected of carrot seeds even for the same accession [3, 5, 17, 45, 46]. Indeed, such was observed in our initial screens of carrot seed lots, with notable differences in germination rates in water, with no abiotic stresses present. We used a robust experimental setup of the assays to assess the salinity and drought tolerance of various carrot accessions. Significant variations in germination rates were observed among different genotypes under both stress conditions, which indicated genotype-specific responses to salinity and drought. But, the breeding line M1945 and commercial cultivar ‘Perfekcja’ analyzed in this study present great promise towards future carrots with improved germination characteristics and were thus subjected to additional testing.

Research aimed at comparing breeding lines and commercially available cultivars (some of which are F1 hybrids) under abiotic stresses such as salinity, drought, or temperature extremes can provide valuable insights into the adaptability and resilience of different genotypes. This will later identify specific traits or genes associated with stress tolerance, aiding in the breeding of more resilient carrots. Related studies in other vegetable (tomato, lettuce) and agricultural crops (rice, wheat, maize) convergently noted that salt stress and drought stress have severely affected the agronomic traits by inhibition of seed germination and plant growth, reduction in biomass and final yield, and decreases in harvest quality [47–50].

### Mechanistic insights from metabolic inhibitor studies

Cellular processes that govern mechanisms of stress tolerance can be uncovered by studying the factors that inhibit them or promote them. We leveraged here the broadly accepted role of four inhibitors [13, 24, 51–53], to analyze how each of them impact carrot germination in water or under imposed drought or salinity stress. Because no major germination decreases in water were observed for any of three studied carrot accession and none of the four inhibitors, their effects observed under imposed salinity or drought can be disentangled as directly influencing the tolerance levels. Effects of these inhibitors on seed germination vary in various studied species. This could be because of the storage polymers specific to given species or genotype, experimental conditions such as method of stress imposition and its severity, inhibitor concentration or time of exposure, or priming pre-treatment [13, 24, 51, 53]. An alternative approach requires a comparatively more in-depth knowledge of specific promoters for any studied cellular processes, with very scant experimental data to that effect available for carrots. Detailed subsequent studies of hybrid progenies of sensitive × tolerant carrots will enable identification of genes and molecular mechanisms -and thereby possible biochemical switches- to experimentally validate such potential germination enhancers.

Use of four inhibitors in our assays implies that the germination of carrot seeds under salinity or drought stress is influenced by specific cellular processes. The inhibition of germination by these inhibitors under abiotic stress conditions suggests that the decline in germination is due to disruptions in specific cellular and metabolic processes. Notably, the unexpected activation of germination under salinity by certain inhibitors highlights the complexity of regulatory mechanisms that regulate seed germination under stress conditions. For instance, the application of cycloheximide, an inhibitor of protein synthesis, resulted in increased germination rates under salinity stress. This suggests that certain stress-responsive proteins may act as negative regulators of germination under salinity, and their inhibition can alleviate this suppression. Indeed, cycloheximide has been confirmed to decrease germination of primed rapeseed or grasses [13, 52]. But, under stress conditions, some non-affected germination was observed after cycloheximide treatment in cucumber or clover [23, 24]. This inhibitor specifically affects the activity of amylases, and as such may variably impact seeds of species with diverse starch pools as storage polymers.

Conversely, actinomycin D, an inhibitor of transcription, significantly reduced germination under salinity stress, indicating that active transcription of stress-responsive genes is crucial for germination under these conditions. These findings underscore the importance of disruption of essential cellular processes, specifically transcriptional and translational regulation, in mediating stress responses during seed germination. The effects of these inhibitors can vary depending on the specific plant species and environmental conditions, and, therefore, further research is needed to fully understand their effects on seed germination under various conditions [12, 13, 54]. Many other factors can influence seed germination; these include the presence of natural germination inhibitors in the seed or its environment, and various abiotic stresses such as salinity, drought, or temperature extremes [5, 55–57]. Understanding these factors can help in the development of strategies to improve seed germination and crop productivity under changing environmental conditions [1, 12, 58]. Future research on the role of phytohormones (e.g., abscisic acid, gibberellins, ethylene) in regulating seed germination and stress responses is important for identification of hormonal pathways involved in stress responses that can lead to targeted interventions and enhanced seed germination under adverse conditions [1, 9, 47].

### Seed priming and stress tolerance

Seed priming can increase seed nutrient content and improve seed quality for improved germination, seedling establishment, plant growth, nutrient uptake, and water use efficiency of plant crops [14, 15]. The mechanisms by which seed priming enhances abiotic stress tolerance include the activation of antioxidant enzymes, accumulation of osmoprotectants, and upregulation of stress-responsive genes. These mechanisms help in mitigating oxidative damage, maintaining cellular homeostasis, and enhancing overall stress resilience. Our seed priming experiments aimed to analyze the effectiveness of seed germination and revealed that pre-treatments with various substances (i.e., hydropriming, osmopriming, biopriming) did not significantly affect the outcomes of germination under control or stress conditions. The priming agents are known to enhance stress tolerance by mechanisms such as improving water uptake, enhancing membrane stability, and activating stress-related signaling pathways. But, significant differences in germination rates were observed under different stress conditions, underscoring the importance of environmental factors in seed germination. Here, the imposed salinity resulted in relatively stronger germination decreases than the drought treatment, with a markedly broad range of germination responses regardless of the priming substance used. The differential effectiveness of priming agents suggests that they may activate distinct physiological and biochemical pathways that confer stress tolerance. For instance, priming with NaCl likely induces osmotic adjustment mechanisms, whereas PEG priming may enhance water uptake efficiency and cellular hydration. The observed improvements in germination and seedling growth following priming treatments highlight the potential of seed priming as a practical strategy to enhance crop resilience to abiotic stresses. Identification of the most effective priming techniques can improve germination rates and seedling vigor, thus enhancing crop establishment in adverse conditions. In other crops systems, seed priming increased the chilling tolerance of rice seeds during germination and growth by enhancing α-amylase activity and soluble sugar content [59]. Summary of successful reports of priming highlighted the remarkable results in terms of growth, yield, disease resistance, abiotic and biotic stress tolerance [22]. A meta-analysis of seed priming effects on seed germination, seedling emergence, and crop yield indicated that seed priming influences germination (rate or percentage), seedling emergence (rate or percentage) and crop yield [60]. Indeed, priming improves seed germination under adverse conditions [61]. Effects of seed priming on germination and subsequent plant growth under various conditions yields the potential as a strategy to enhance crop establishment and productivity [62–64].

### Seedling growth under abiotic stresses

Analyses of seedling growth dynamics provided insights into the impact of salinity and drought on the initial stages of root and shoot development. Significant interactions between genotype, germination conditions, and duration of growth post-imbibition highlighted the complexity of factors influencing seedling growth in carrot, but the sensitive accession scored worse than the tolerant one regardless of what parameter was compared in this aspect. Research on the effects of drought or salinity on seed germination and early growth stages can help develop irrigation and soil management practices that optimize germination and growth under saline conditions. Investigations into how root architecture and root growth dynamics are affected by abiotic stresses in carrots and other crops (Nowicki et al., unpublished data) can aid in the selection of genotypes with root traits that confer better stress tolerance and water/nutrient uptake efficiency. Similarly, studies on the effects of abiotic stresses on shoot development, including leaf area, biomass accumulation, and photosynthetic efficiency can help in developing strategies to enhance shoot growth and overall plant vigor under stress conditions. Similar related studies provided valuable insights into the adaptability and resilience of different genotypes of various crops under abiotic stresses such as salinity, drought, or temperature extremes. Under drought, steeper root angles and deeper root systems were proposed as advantageous for both maize and sorghum. In saline soils, a reduction in root length and root number has been described as advantageous [65]. Reduction in germination potential, early seedling growth, root and shoot dry weight, hypocotyl length, and vegetative growth have been reported in important field crops including, pea (*Pisum sativum* L.), alfalfa (*Medicago sativa* L.), and rice (*Oryza sativa* L.) under drought stress [66]. Similarly, drought stress led to a decline in germination percentage, accompanied by variable changes in root length, shoot length, root/shoot ratio, root numbers, and biomass in tomato, onion, and cucumber [18–20, 67].

### Biochemical responses and genotype-specific adaptations

We also investigated the involvement of reactive oxygen species (ROS) levels, detoxifying enzymes, and protective metabolites in the response of carrot seedlings to salinity and drought stress. Understanding the physiological and biochemical responses can help develop strategies to mitigate stress effects, such as through the application of bio-stimulants or stress ameliorants. Some of these responses were common across all the carrot accessions tested here, others were genotype-specific, thus indicating adaptations to stress conditions at the biochemical level. The observed biochemical responses, such as the decreases in SOD activity under salinity stress and the specific increase in POX activity under drought in certain accessions, underscore the importance of genotype-specific adaptations in conferring salinity and drought tolerance in carrot seedlings. Our biochemical analyses revealed genotype-specific responses in ROS levels and antioxidant enzyme activities, which are critical for managing oxidative stress during germination under abiotic stress conditions. For example, the increase in ROS levels under drought stress and the corresponding activation of antioxidant enzymes such as superoxide dismutase (SOD) and peroxidase (POX) in tolerant genotypes like M1945 and ‘Perfekcja’ suggest that these genotypes possess robust antioxidant defense mechanisms. These mechanisms likely mitigate oxidative damage and support cellular homeostasis, thereby enhancing germination and seedling vigor under stress. The observed decrease in SOD activity under salinity stress across all genotypes indicates a common response to salinity, potentially reflecting a shift in the balance between ROS production and scavenging.

### Future directions and conclusions

This research is innovative in its comprehensive approach to understanding the mechanisms of seed germination and seedling development under abiotic stress conditions. By integrating seed priming techniques with the application of cellular process inhibitors and biochemical analyses, this study provides a multi-faceted view of how carrot seeds respond to salinity and drought stress. The findings contribute to the in-depth understanding of stress tolerance mechanisms in plants, highlighting the roles of transcriptional and translational regulation, antioxidant defense systems, and osmotic adjustment. This research not only advances our knowledge of carrot physiology but also offers practical strategies for improving crop resilience, which can be applied to other crops facing similar environmental challenges. Ongoing and future investigations of the molecular mechanisms that orchestrate the biochemical responses to salinity or drought (genotyping-by-sequencing; Genome-Wide Association Studies; RNAseq; epigenomics) may add additional insights into the underpinnings of carrot tolerance to abiotic stresses. Integrative studies using omics approaches (e.g., transcriptomics, proteomics, metabolomics) to elucidate the molecular pathways involved in stress responses can lead to the identification of biomarkers for stress tolerance and the development of stress-resilient crops. Investigations into the roles of phytohormones (e.g., abscisic acid, gibberellins, ethylene) in regulating seed germination and stress responses will provide deeper insights into the hormonal regulation of stress tolerance. Investigations into how ROS and antioxidant systems mediate seed germination and early seedling growth under stress conditions can provide valuable data for modulating ROS levels and antioxidant defenses as a strategy to improve stress tolerance during germination. Whereas, studying the roles of various antioxidant enzymes and non-enzymatic antioxidants in mediating stress tolerance in carrots and other crops can help identify key components of the antioxidant defense system that could be targeted for enhancing stress tolerance. Additionally, field trials are essential to validate the effectiveness of identified tolerant accessions and treatments in real-world agricultural settings. Comparative studies with other crops can identify common stress tolerance mechanisms and potential cross-species applications, leading to broader agricultural applications and collaborative efforts in crop improvement programs. Implications of multiple stresses on vegetable crops may vary from those on a single stress [68]. Stress-tolerant cultivars are being developed using a variety of methods, including traditional breeding and transgenic technology. Instead of genetic engineering, using vegetable breeding procedures or directed breeding is one the best options to improve stress tolerance in vegetables [69].

In summary, our study provides valuable insights into the mechanisms underlying salinity and drought tolerance in carrot seedlings and contributes to our understanding of plant stress responses. The practical recommendation that stems from this study is that seed pretreatment is a simple valuable method to limit the carrot losses at germination stage under the inevitable abiotic stresses. Our carrot germplasm screening also provides a platform for possible high-throughput method of carrot germplasm selection, as well as information about at least two carrot accessions (M1945; ‘Perfekcja’) well-suited for use with regards to their salinity and drought responses. Future field trials are essential to confirm the effectiveness of identified tolerant accessions and treatments in actual agricultural settings. Similarly, an assessment of the cost-benefit ratio and potential yield improvements can help in the practical adoption of our findings by farmers. Beyond carrot, we studied under similar setup accessions of cabbages, tomatoes, and cucumbers (Nowicki et al., unpublished data). Comparative studies with these and other crops to identify common stress tolerance mechanisms and potential cross-species applications can lead to broader agricultural applications and collaborative efforts in crop improvement programs. Finally, anticipation of future climatic changes can guide breeding programs and management practices to develop more resilient crops.

## Supporting information

S1 Fig Seeds of M1939, M1945, and ‘Perfekcja’ germinating on water, NaCl, KCl, NPK, and PEG. Pear each plate, 25 seeds for each carrot accession were plated out and recorded at 4 days post-imbibition (dpi), 5 dpi, and 6 dpi in 10 cm square Petri dishes lined up with Whatman filter paper soaked with 5 mL of the respective solution.
(TIF)

S2 Fig Visualization of 7-day-old seedlings of M1939, M1945, and ‘Perfekcja’ grown under various conditions (water; NaCl; PEG). The seedlings are shown with a 1 cm scale for reference.
(TIF)

S1 Table Results of one-way, two-way, or three-way ANOVA and post-hoc HSD tests for various experimental conditions. This table summarizes the results of one-way, two-way, or three-way ANOVA analyses followed by post-hoc Tukey’s Honest Significant Difference (HSD) tests for multiple experimental conditions. Each section of the table corresponds to a specific test, indicated by the test name (e.g., "Germination in water"), followed by reference to the specific Main Text figure (e.g., [Fig 1]). For each test, the ANOVA summary includes the degrees of freedom (Df), sum of squares (Sum Sq), mean square (Mean Sq), F-value, and p-value (Pr(>F)). The post-hoc HSD test results list the groups compared, their mean values, and the significance groupings, where groups sharing the same letter are not significantly different from each other at the 0.05 significance level.
(XLSX)
