# Supplementary figures and images for: Seed priming and abiotic stress tolerance in carrot: Unraveling the mechanisms of improved germination
Source: PLoS One. 2025 Feb 7;20(2):e0318753. doi: 10.1371/journal.pone.0318753 (PMC11805430; doi:10.1371/journal.pone.0318753)

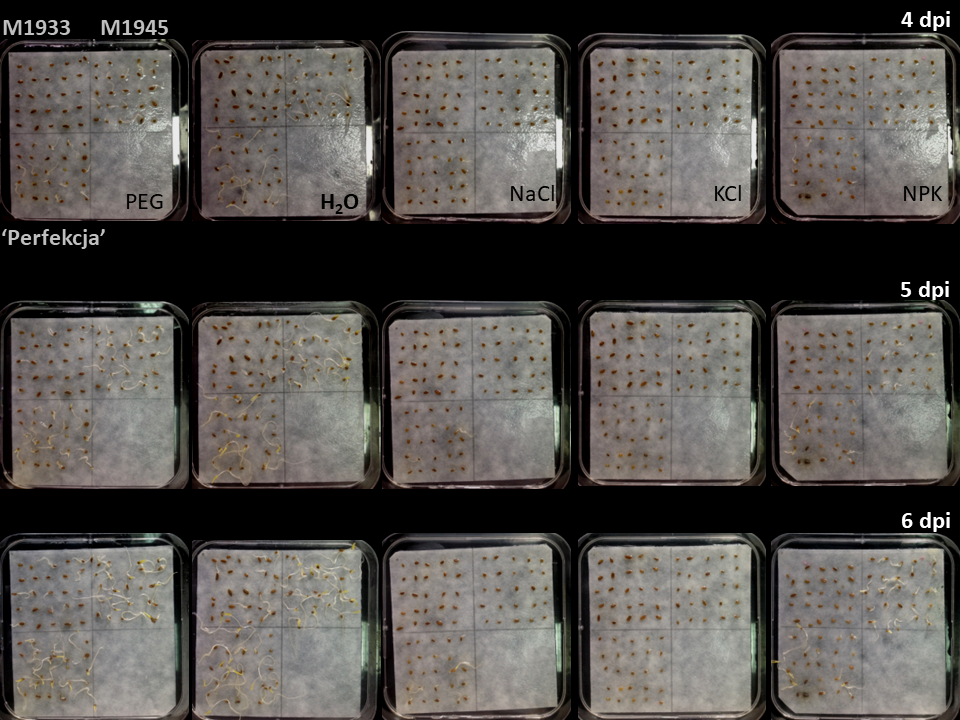

Supplement: S1 Fig — Pear each plate, 25 seeds for each carrot accession were plated out and recorded at 4 days post-imbibition (dpi), 5 dpi, and 6 dpi in 10 cm square Petri dishes lined up with Whatman filter paper soaked with 5 mL of the respective solution. (TIF) [file pone.0318753.s001.tif]

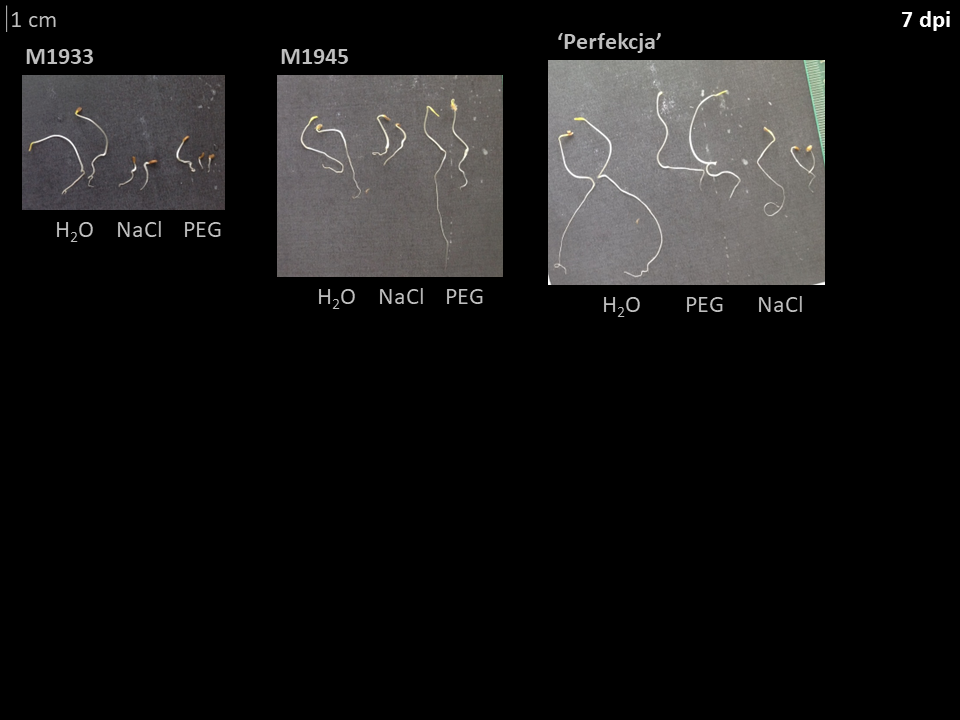

Supplement: S2 Fig — The seedlings are shown with a 1 cm scale for reference. (TIF) [file pone.0318753.s002.tif]
